# Glucose Homeostasis in Mice Is Transglutaminase 2 Independent

**DOI:** 10.1371/journal.pone.0063346

**Published:** 2013-05-22

**Authors:** Siiri E. Iismaa, Mark Aplin, Sara Holman, Ting W. Yiu, Kristy Jackson, James G. Burchfield, Christopher J. Mitchell, Liam O’Reilly, Aimee Davenport, James Cantley, Carsten Schmitz-Peiffer, Trevor J. Biden, Gregory J. Cooney, Robert M. Graham

**Affiliations:** 1 Molecular Cardiology and Biophysics Division, Victor Chang Cardiac Research Institute, Sydney, New South Wales, Australia; 2 Diabetes and Obesity Division, Garvan Institute of Medical Research, Sydney, New South Wales, Australia; 3 University of New South Wales, Sydney, New South Wales, Australia; Univeristy of California Riverside, United States of America

## Abstract

Transglutaminase type 2 (TG2) has been reported to be a candidate gene for maturity onset diabetes of the young (MODY) because three different mutations that impair TG2 transamidase activity have been found in 3 families with MODY. TG2 null (TG2^−/−^) mice have been reported to be glucose intolerant and have impaired glucose-stimulated insulin secretion (GSIS). Here we rigorously evaluated the role of TG2 in glucose metabolism using independently generated murine models of genetic TG2 disruption, which show no compensatory enhanced expression of other TGs in pancreatic islets or other tissues. First, we subjected chow- or fat-fed congenic SV129 or C57BL/6 wild type (WT) and TG2^−/−^ littermates, to oral glucose gavage. Blood glucose and serum insulin levels were similar for both genotypes. Pancreatic islets isolated from these animals and analysed *in vitro* for GSIS and cholinergic potentiation of GSIS, showed no significant difference between genotypes. Results from intraperitoneal glucose tolerance tests (GTTs) and insulin tolerance tests (ITTs) were similar for both genotypes. Second, we directly investigated the role of TG2 transamidase activity in insulin secretion using a coisogenic model that expresses a mutant form of TG2 (TG2^R579A^), which is constitutively active for transamidase activity. Intraperitoneal GTTs and ITTs revealed no significant differences between WT and TG2^R579A/R579A^ mice. Given that neither deletion nor constitutive activation of TG2 transamidase activity altered basal responses, or responses to a glucose or insulin challenge, our data indicate that glucose homeostasis in mice is TG2 independent, and question a link between TG2 and diabetes.

## Introduction

Type 2 diabetes mellitus (T2DM) is characterised by defects in both end-organ responsiveness to insulin (insulin resistance) and the regulation of insulin release by pancreatic β cells. A variant is maturity-onset diabetes of the young (MODY), a monogenic form of the disease responsible for 1–2% of T2DM [1], [2]. Although causal mutations in several genes (e.g. HIF-1α, HNF-4α) have been identified, others remain unknown. Transglutaminase type 2 (TG2) has been reported to be a candidate gene for MODY, with three types of missense mutations found in the TG2-encoding gene (TGM2) in 3 families with early-onset Type 2 diabetes [3], [4].

TG2, also known as tissue transglutaminase or G_h_ (high molecular weight GTP-binding protein), is a multifunctional protein. Two independently-generated TG2 null mouse models [5], [6] have demonstrated TG2 involvement in diverse intra- and extracellular pathophysiological processes, including cataract development, gluten sensitivity diseases, neurodegeneration, and tissue remodelling/repair associated with heart, liver, and kidney disease, cancer and bone development [7]. TG2 has three major biological activities: *first*, as a calcium-activated protein-crosslinking enzyme, it has transamidase activity, which results in linkage between intrachain glutamine and lysine residues to form an Nε-(γ-glutamyl) isopeptide bond, incorporation of an amine onto a glutamine residue, or acylation of a lysine residue [8]; s*econd*, TG2 binds and hydrolyses GTP [9], [10]. GTP binding inhibits transamidase activity, enabling TG2 to function as an intracellular G-protein that mediates signalling by various G-protein coupled receptors (GPCRs), including α_1B_ and α_1D_-adrenergic [11]–[13], thromboxane A2 [14], [15] and oxytocin [16] receptors, to activate phospholipase C (PLC); *third*, TG2 functions as an extracellular adaptor protein that facilitates interaction between the matrix and β1/β3-integrins to promote fibroblast adhesion and spreading [17]. The transamidase activity of TG2 has been suggested to modulate the exocytosis step of glucose-stimulated insulin secretion (GSIS) that accompanies calcium influx into pancreatic β cells [18]–[20]. Consistent with TG2 involvement in insulin release, pancreatic islets from TG2 null mice [5] have been reported to secrete less insulin, relative to wild-type (WT) islets, in response to a high glucose challenge [3], [21]. This was reflected in lower blood insulin and higher blood glucose levels in TG2^−/−^ mice following intraperitoneal glucose loading [3].

Regulation of insulin secretion from pancreatic β cells is complex, involving direct responses to nutrients as well as amplification of these signals by gut-derived peptides and neural mechanisms; the latter involving acetylcholine released from vagal efferents acting on β cell muscarinic GPCRs of the M1 and M3 subtype [22]–[24]. Muscarinic receptors are G-protein-coupled: M1, M3, and M5 subtypes activate PLC/protein kinase C (PKC) signalling pathways via G_q_ and/or G_11_, and M2 and M4 subtypes inhibit adenylyl cyclase via G_i_. Given that TG2, like G_q_/G_11_, can function as a G-protein for signalling by a number of GPCRs that activate PLC, we investigated the role of TG2 in mediating muscarinic modulation of insulin release and glucose homeostasis. Our results, using two different congenic TG2 null mouse lines (generated in this work from mixed strain TG2^−/−^ mice reported by us [6]) as well as a coisogenic mouse line constitutively active for TG2 transamidating activity (generated in this work), indicate no role for TG2 in either glucose homeostasis or muscarinic modulation of glucose homeostasis. This suggests, therefore, that glucose homeostasis is TG2 independent and questions a role for TG2 in the pathophysiology of T2DM.

## Materials and Methods

### Ethics Statement

All experimental procedures were approved by the Garvan Institute/St. Vincent’s Hospital Animal Experimentation Ethics Committee (No. 04/08, 07/12, 09/30) and were performed in strict accordance with the National Health and Medical Research Council (NHMRC) of Australia Guidelines on Animal Experimentation. All efforts were made to minimize suffering.

### Mice

Heterozygous TG2^+/−^ mice (Tgm2^tm1.1Rmgr^) originally on a mixed C57BL/6J–129S1/SvImsJ strain (designated B6;129) background [6], were backcrossed to either C57BL/6J or 129T2/SvImsJ mice for twelve generations to generate congenic heterozygous TG2^+/−^ mice with 99.95% C57BL/6J (B6.129-Tgm2^ tm1.1Rmgr^; designated B6 WT or TG2^−/−^) or 129T2/SvImsJ (129.Cg-Tgm2^tm1.1Rmgr^; designated 129 WT or TG2^−/−^) genomic homogeneity, respectively. B6 or 129 TG2 null lines were routinely maintained as heterozygous breeding pairs and backcrossed after every second or third generation. Heterozygotes were crossed to generate congenic TG2^+/+^ (WT) and TG2^−/−^ littermates. PCR genotyping of DNA from ear clippings involved two separate reactions: one using primers KOFP1 (5′-GGAGCACACAGGCCTTATGAGCTGAAG-3′; complementary to an intronic region between exons 5 and 6) and RP3 (5′-GCCCCACAAAGGAGCAAGTGTTACTATGTC-3′; reverse complement of an intronic region 3′ of the remnant loxP site that is located between exons 8 and 9) to yield a 300 bp product from the knock-out allele and the other using primers WTFP2 (5′-CAGATAGGGATACAAGAAGCATTGAAG-3′; complementary to an intronic region 5′ of the loxP site that is located between exons 5 and 6) and RP3 to yield a 100 bp product from the WT allele.

A TG2^R579A^ knock-out/knock-in mouse that has Ala substituted for Arg at position 579 of TG2 was generated by OzGene (Australia) on a C57BL/6J background (B6-Tgm2^tm2Rmgr^; designated B6 WT or TG2^R579A/R579A^). The construct used for homologous recombination into C57BL/6J (Bruce4) embryonic stem cells (ES) encompassed exons 10–13 of *Tgm2* (with ∼6kb of DNA homology on either side of codon 579). The Arg^579^ codon in exon 11 (AGA) was mutated to Ala (GCC) thereby removing a *Bgl*II restriction enzyme site. A new restriction enzyme site (*Nhe*I) was introduced by silent mutation of Leu^575^ (CTG to CTA) and a *loxP*-flanked phosphoglycerate kinase (PGK) promoter–neomycin resistance–polyA cassette was inserted between exons 11 and 12. Neomycin-resistant homologously-recombined Bruce4 ES cells were identified by Southern analysis and injected into Balb/c blastocysts. High-percentage chimeras were crossed with C57BL/6J mice to generate germline WT/TG2^R579A^+Neo offspring. These mice were crossed with a C57BL/6J homozygous Oz-Cre deleter strain to remove the PGK–neomycin selection cassette and generate WT/TG2^R579A^ΔNeo/Cre offspring. These mice were crossed with C57BL/6J to generate heterozygous WT/TG2^R579A^ΔNeo/ΔCre offspring. This line was routinely maintained as heterozygous breeding pairs and backcrossed after every second or third generation. Heterozygotes were crossed to generate co-isogenic TG2^+/+^ (WT) and TG2^R579A/R579A^ littermates. Genomic DNA sequencing confirmed the TG2^R579A^ mutation in the first homozygotes generated. PCR genotyping of DNA from ear clippings used primers KIFP1 (5′-TACGAGAAGTACAGCGGGTGCCTGACA-3′; complementary to an exon 11 region upstream of codon 579) and KIRP2 (5′-AATGCTTTCCACAAGGACCCAGAGAC-3′; reverse complement of an intronic region 3′ of the remnant loxP site) to yield a 250 bp *Bgl*II-cleavable product from the WT allele and a 320 bp *Nhe*I-cleavable product from the knock-in allele. Detailed analysis of the phenotype of this line will be presented elsewhere.

### Diets

Mice were housed at an ambient temperature of 19–23°C, (mean temp 22°C) under a 12 h dark/12 h light cycle in sterilized cages with PuraChip Coarse Dust free Aspen bedding (Able Scientific) and fed a standard (chow) diet (Rat and Mouse Premium Breeder Diet: 23% protein, 6% fat, 5% fibre; irradiated (25 kgy), Gordon’s Specialty Stockfeeds, Australia) with *ad libitum* water. For dietary studies, 3 month-old male mice were randomly divided into two diet groups and maintained for 3 months on a chow or high-fat diet [25] as indicated. The high fat diet consisted of 23% w/w casein (acid casein, MPD Dairy), 20.2% w/w sucrose (Cat. No. GRAD25B, JL Stewart), 17% w/w starch (Cat. No. CFLR2M, JL Stewart), 4.5% w/w homemade mineral mix (0.0014% w/w NaSeO_4_, Cat. No. S0882 Sigma; 0.001% w/w KIO_3_, Cat. No. 207977 Sigma; 0.055% w/w CrK(SO_4_)_2_.12H_2_O, Cat. No. 243361 Sigma; 0.063% w/w MnCO_3_, Cat. No. 306 Ajax; 0.498% w/w FeSO_4_.7H_2_O, Cat. No. F7002 Sigma; 0.16% w/w ZnCO_3_.2ZnO.3H_2_O, Cat No. 1518 Ajax; 0.03% w/w CuCO_3_.Cu(OH)_2_, Cat No. 207896 Sigma; 13.488% w/w starch, Cat. No. CFLR2M, JL Stewart; 35.671% w/w CaCO_3_, Cat. No. 102059 Merck Millipore; 40.209% KH_2_PO_4_, Cat. No. 104873 Merck Millipore; 7.4% w/w NaCl, Cat. No. S9625 Sigma; 2.4% MgO, heavy, Cat. No. 835, Ajax), 1.3% w/w trace minerals (0296026401, MP Biomedicals), 5% w/w bran (BRANIOUF, JL Stewart), 0.3% w/w methionine (M9500, Sigma), 2% w/w gelatine (GELA2, JL Stewart), 0.4% w/w choline bitartate (C1629, Sigma) stored as a dry powder with 3% w/w safflower oil (311964001790, Proteco Gold), 22% w/w copha (Fonterra), and 1.3% w/w AIN76A vitamins (960098, MP Biomedicals) added on the day.

### Glucose Tolerance Tests

For intraperitoneal or oral glucose tolerance tests, mice were fasted 6 h or overnight (16 h) before glucose (2 g/kg body weight) administration by intraperitoneal injection or gavage, respectively. Tail vein blood glucose was measured (Accu-Chek® Performa glucometer) at 0, 15, 30, 45, 60, 90, and 120 min after injection. Whole blood (50 µl) was collected at 0 and 15 mins, and serum was stored at −80°C for later analysis of insulin levels using a radioimmunoassay specific for rodent insulin (Linco Research Immunoassay, USA).

### Insulin Tolerance Tests

For insulin tolerance tests, mice were fasted for 5–6 hours, then injected intraperitoneally with insulin (0.75 U/kg body weight) at 12∶00–14∶00 h and tail vein blood glucose was measured as above at 0, 15, 30, 45 and 60 min after injection.

### Islet Isolation and Insulin Secretion Assays

Mice were euthanized by cervical dislocation following anaesthesia with 5% isoflurane and the common bile duct was cannulated and its duodenal end occluded by clamping. Two ml of Liberase (Roche, Basel, Switzerland) solution (0.25 mg/ml in M199) were injected into the duct to distend the pancreas [26]. The pancreas was excised, incubated at 37°C for 17 min, and mechanically disrupted in 10 ml M199. Cellular components were collected by density centrifugation (130 g for 2 min), washed and re-suspended in 10 ml RPMI1640/10% FCS. Islets were hand-picked under a microscope, washed once in Krebs-Ringer bicarbonate (KRB) buffer pH 7.4 and cultured overnight in RPMI1640/10% FCS with 11 mM glucose.

For insulin secretion studies of batch incubations, islets were pre-incubated for 1 h in 1 ml KRB buffer (supplemented with 0.25% BSA and 10 mM HEPES) containing 2.8 mM glucose and insulin release was assessed after a static 1 h incubation at 37°C/5% CO_2_ of groups of five islets, size-matched by handpicking, in KRB buffer containing 2.8 mM glucose, 16.8 mM glucose, or 16.8 mM glucose plus 0.1 mM carbachol. Islets for perifusion studies were perifused as described [27] at 37°C at 0.5 ml/min with 2.8 mM glucose in KRB buffer for 20 min before experimental additions. Secreted insulin from batch supernatants and perifusates was assayed by radioimmunoassay with rat insulin standard (Linco Research, USA). Total cellular insulin islet content was extracted with acid-ethanol (1.5% HCl in 75% ethanol) and assayed by RIA.

### Reverse-Transcription Quantitative Real-Time PCR (RT-qPCR) Analysis

RNA was isolated from tissues of TG2^+/+^, TG2^−/−^ or TG2^R579A/R579A^ mice (n = 3) using the miRNeasy RNA extraction kit (Qiagen, Germany). RNA integrity was measured by RNA 6000 Nano Chip using Agilent Bioanalyzer 2100 (Agilent Technologies, USA) and reverse transcription performed using Superscript III reverse transcriptase (Invitrogen, Australia). RT-qPCR was performed in triplicate using the Taqman Gene Expression Assay 384 well format (Applied Biosystems, Australia). Expression levels of *Tgm1* (assay ID: Mm00498375_m1*, amplicon length 83 bp spanning exons 9–10, Taqman probe sequence: 5′-GACCAGCAGTGGCATCTTCTGCTGT-3′ with anchor nucleotide location at 1512 of NM_001161714.1), *Tgm2* (Mm00436987_m1*, amplicon length 72 bp spanning exons 10–11, Taqman probe sequence: 5′-CCTGGATCCCTACTCTGAGAACAGC-3′ with anchor nucleotide location at 1700 of NM_009373.3), *Tgm3* (Mm00436999_m1*, amplicon length 61 bp spanning exons 11–12, Taqman probe sequence: 5′-CCTGCTTTGACCCTGGAGGTGCTGG-3′ with anchor nucleotide location at 1824 of NM_009374.2), *Tgm4* (Mm00626039_m1*, amplicon length 71 bp spanning exons 10–11, Taqman probe sequence: 5′-AGTTCCCAGAAGGCAGCCCAGAGGA-3′ with anchor nucleotide location at 1358 of NM_177911.4), *Tgm5* (Mm00551335_m1*, amplicon length 59 bp spanning exons 11–12, Taqman probe sequence: 5′-TGATTAATGTTCTAGGAGCTGCCTT-3′ with anchor nucleotide location at 1980 of NM_028799.2), *Tgm6* (Mm00624922_m1*, amplicon length 63 bp spanning exons 5–6, Taqman probe sequence: 5′-TCATCAGTGCCATGGTGAACAGCAA-3′ with anchor nucleotide location at 748 of NM_177726.3), Tgm7 (Mm03990491_m1*, amplicon length 61 bp spanning exons 5–6, Taqman probe sequence: 5′-CTGTTATGTGCACGGTAATGAGATG-3′ with anchor nucleotide location at 863 of NM_001160424.1) and *F13a1* (Mm00472334_m1*, amplicon length 62 bp spanning exons 14–15, Taqman probe sequence: 5′-AGAAAGGTGTTCCGTGAAATCCGGC-3′ with anchor nucleotide location at 2112 of NM_001166391.1) were quantitated against Hprt (Mm00446968_m1*, amplicon length 65 bp spanning exons 6–7, Taqman probe sequence: 5′-GTTAAGGTTGCAAGCTTGCTGGTGA-3′ with anchor nucleotide location at 630 of NM_013556.2) as the most suitable reference gene. A standard curve using TG2^+/+^ cDNA diluted to 1, 1∶10, 1∶100, and 1∶1,000 was constructed and a cDNA negative reaction were included for each gene examined. Results were only accepted if the crossing point was between 20–30 cycles and if the standard deviation within one triplicate was less than 0.5.

### Immunoblotting

TG2 expression levels were evaluated in various tissues by Western blotting using anti-mouse TG2 rat monoclonal antibody (a gift from Gail V. W. Johnson, University of Rochester, NY, USA; 1∶16,000 dilution in Tris-buffered saline for 1 h at room temperature) and quantitated against either GAPDH (Santa Cruz, USA) or β-tubulin (Sigma, USA) as a loading control.

### Transamidase Assays

*In vitro* calcium-activated transamidase activity [28] of liver lysates (100 µg) and intracellular transamidase activity [29] of murine embryonic fibroblasts were assayed. The relative insensitivity of the intracellular transamidase assay coupled with non-homogeneous diffusion of 5-(biotinamido)pentylamine into islets precluded measurement of intracellular islet transamidase activity.

### Statistical Analyses

Data are expressed as means ± SEM. Blood glucose and serum insulin levels in congenic or co-isogenic strains, qPCR and intracellular transamidase activity were compared using 2-way ANOVA with the Bonferroni post hoc test; body weights of congenic or co-isogenic strains and *in vitro* transamidase activity of lysates were compared using 1-way ANOVA with the Bonferroni post hoc test (GraphPad Prism). The level of statistical significance was set at *P*<0.05.

## Results

### No Compensation by Other TGs in Congenic TG2 Null Lines

**Figure 1:**
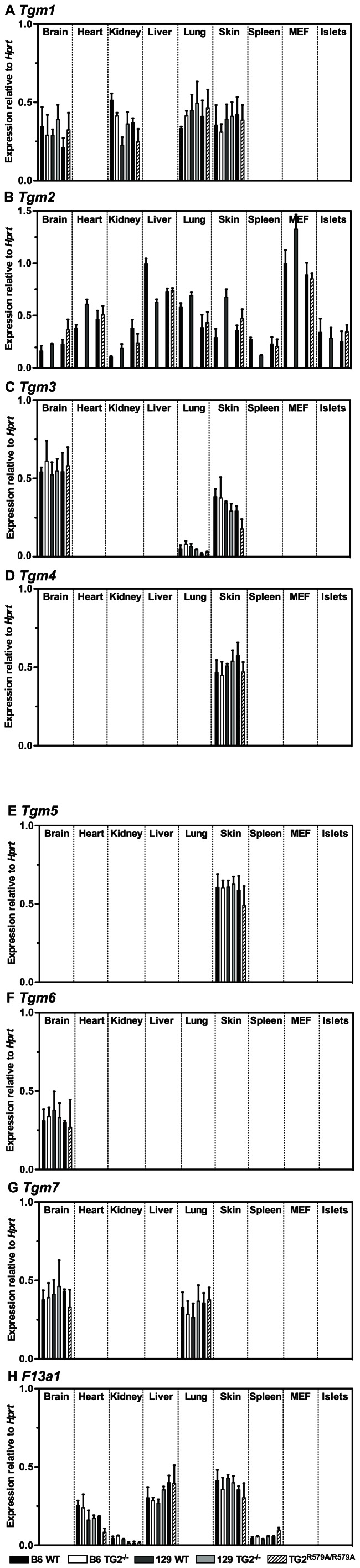
Neither *Tgm2* deletion nor substitution with *Tgm2^R579A^* enhances expression of other TGs. Brain, heart, kidney, liver, lung, skin, spleen, embryonic fibroblasts and islet mRNA from 3 month-old B6 WT and TG2^−/−^, 129 WT and TG2^−/−^ or B6 WT and TG2^R579A/R579A^ mice was subjected to RT-qPCR analysis of *Tgm1-7* and *F13a1* mRNA expression relative to HPRT mRNA. Data are presented as means ± SEM (n = 3). Statistical significance was calculated using 2-way ANOVA with the Bonferroni *post hoc* test.

Using RT-qPCR analysis of mRNA from brain, heart, kidney, liver, lung, skin, spleen, embryonic fibroblasts and islets (Fig. 1) from B6 or 129 WT and TG2^−/−^ littermates, we found an absence of *Tgm2* mRNA in TG2^−/−^ samples, as expected, and no increase, relative to WT, of *Tgm1*, *Tgm3-7* or *F13a1* mRNA expression in response to TG2 ablation. This indicates that, in the tissues examined, neither congenic TG2 null line compensates for the lack of TG2 by enhancing the expression of other TGs. Strikingly, the only TG expressed in significant quantities by islets is TG2 (Fig. 1). This indicates that any phenotype observed in these mice reflects the role of the deleted *Tgm2* gene.

### Oral Glucose Tolerance Tests in WT and TG2^−/−^ Mice

To investigate the role of TG2 in mediating muscarinic modulation of glucose homeostasis, we first undertook oral glucose tolerance tests. To amplify metabolic defects associated with T2DM, such as impaired insulin secretion [30] or development of insulin resistance [31], 3 month-old congenic male B6 or 129 WT and TG2^−/−^ littermates were fed a high-fat diet for 3 months. The impact of TG2 deletion on glucose metabolism was assessed in these fat-fed animals relative to 6 month-old congenic male B6 or 129 WT and TG2^−/−^ littermates fed a standard chow diet. Within each dietary group, fasting body weight, blood glucose and serum insulin levels of 129 mice were significantly lower (p<0.001) than those of B6 mice (Table 1). Consistent with previous studies [31], B6 mice gained weight on the high-fat diet (mean increase: +16 g) but 129 mice did not (Table 1). Although the average fasting body weights of fat-fed B6 TG2^−/−^ mice was significantly greater (p<0.001) than that of fat-fed B6 WT mice, there was no significant difference in the average fasting body weights between WT and TG2^−/−^ mice on the same congenic background in the other dietary groups (Table 1). Fasting blood glucose and serum insulin levels were also no different between WT and TG2^−/−^ mice on the same congenic background and in the same dietary group (Table 1).

**Table 1:** Fasting body weights (g), blood glucose (mM), serum insulin (ng/ml) and total insulin content per islet (ng/islet) in 6-month old mice fed a normal chow diet (Chow diet) or fed a normal chow diet for 3 months followed by a high-fat diet for 3 months (High-fat diet).

		Chow diet				High-fat diet			
Genetic background	Genotype	Bodyweight	Bloodglucose	Seruminsulin	Islet insulincontent	Bodyweight	Bloodglucose	Seruminsulin	Islet insulincontent
B6	WT	27.9±1.0	8.6±0.3	0.31±0.04	55.3±1.4	41.2±1.4*	10.7±0.8	1.60±0.26*	37.2±4.7
	TG2^−/−^	27.2±0.3	8.2±0.2	0.41±0.07	56.2±3.1	46.3±1.0* #	9.3±0.4	1.66±0.20*	34.8±4.0^x^
129	WT	21.4±1.0‡	4.7±0.2‡	0.14±0.02‡	45.0±1.8	24.1±0.6‡	4.5±0.1‡	0.19±0.03‡	57.0±6.1†
	TG2^−/−^	20.5±1.0‡	4.5±0.4‡	0.11±0.03‡	46.8±2.9	23.7±0.7‡	5.1±0.5‡	0.14±0.02‡	53.5±5.3

Values are presented as means ± SEM (n = 7).

x *P*<0.05 vs chow diet;

* *P*<0.001 vs chow diet;

# *P*<0.001 vs WT;

† *P*<0.05 vs corresponding B6 genotype;

‡ *P*<0.001 vs corresponding B6 genotype.

**Figure 2:**
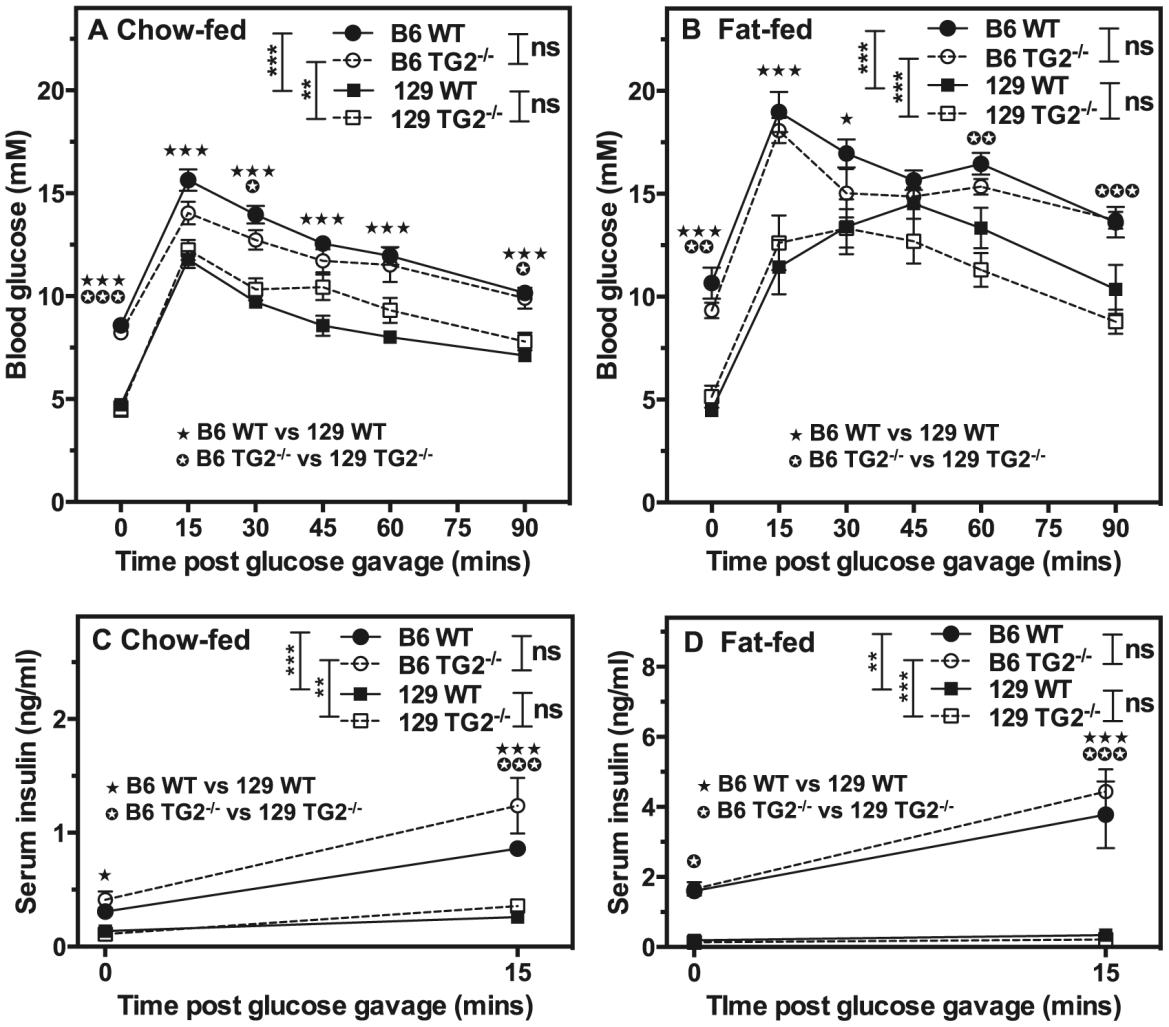
*Tgm2* deletion has no effect on oral glucose tolerance tests in 6 month-old male mice. Male B6 WT and TG2^−/−^ or 129 WT and TG2^−/−^ mice (3 months old raised on a normal chow diet) were fed normal chow for 3 months (A, C) or fed a high-fat diet for 3 months (B, D). Fasted male mice were then subjected to oral glucose tolerance tests, and blood glucose (A, B) and serum insulin (C, D) levels were determined. Data are presented as means ± SEM (n = 7). Overall *P* values calculated using 2-way ANOVA are shown at the top; individual Bonferroni *post hoc* test results are shown above the line profiles. *P<0.05; **P<0.01; ***P<0.001.

To determine whether TG2 might play a more selective role in cephalic phase insulin secretion (including vagal mechanisms) glucose homeostasis was assessed following oral glucose loading. Consistent with previous studies [32], blood glucose levels were significantly higher in male B6 than in male 129 mice following glucose loading (p<0.01 or p<0.001; Fig. 2A&B) and, as expected [31], significantly higher in fat-fed than in chow-fed mice (p<0.001; compare congenic strains in Fig. 2A&B). Similarly, following glucose loading, serum insulin levels were significantly higher in male B6 than in male 129 mice (p<0.01 or p<0.001; Fig. 2C&D) and only in male B6 mice were serum insulin levels significantly higher with fat-feeding than with chow-feeding (p<0.001; compare congenic strains in Fig. 2C&D – note the scale differences in the ordinates of Fig. 2C vs Fig. 2D). Following glucose loading, however, neither blood glucose (Fig. 2A&B) nor serum insulin levels (Fig. 2C&D) were significantly different between WT and TG2^−/−^ mice on the same congenic background and in the same dietary group, indicating no effect of TG2 deletion on muscarinic potentiation of GSIS or insulin action.

### Cholinergic Potentiation of GSIS by WT and TG2^−/−^ β Cells

**Figure 3:**
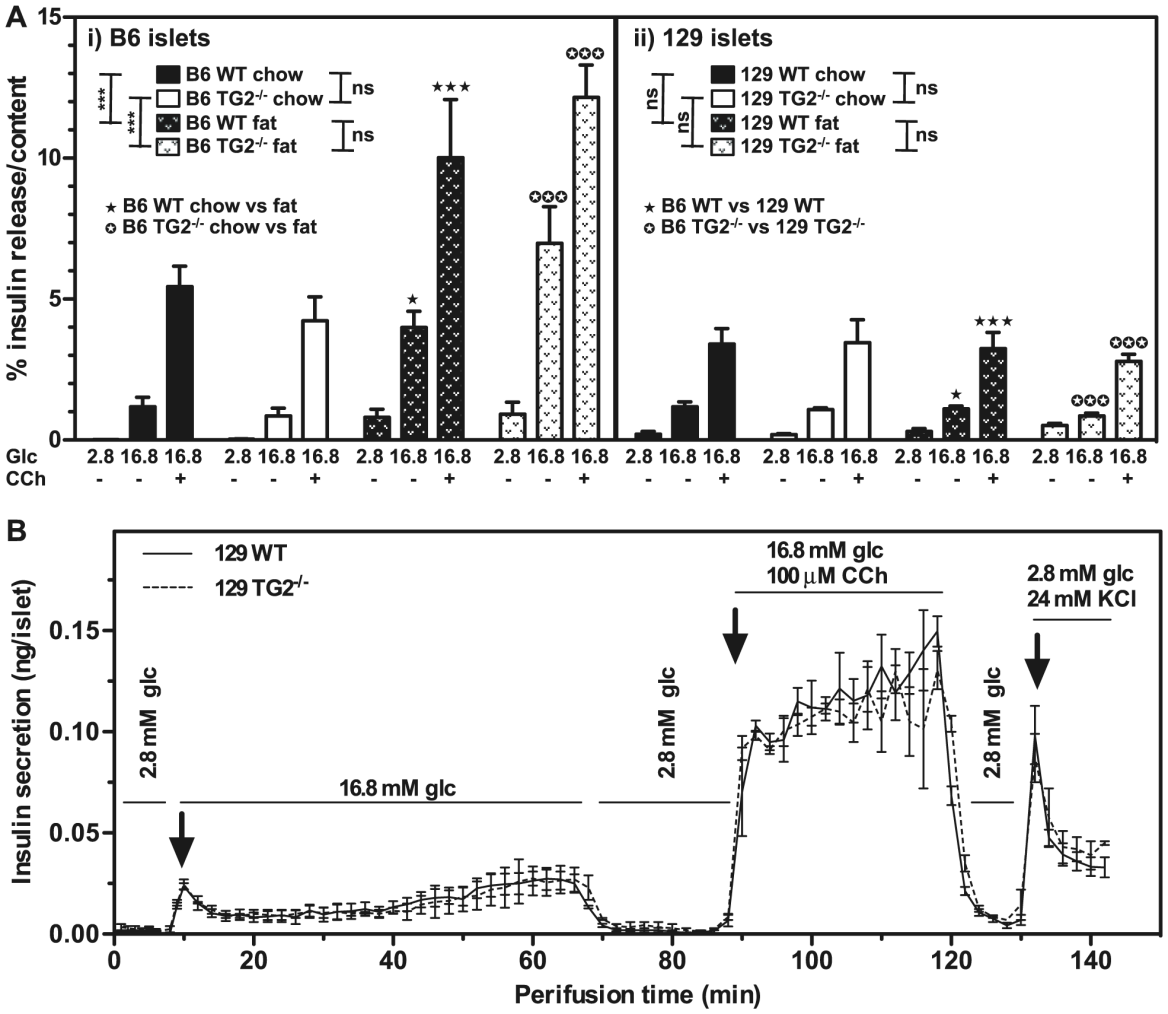
*Tgm2* deletion has no effect on insulin secretion by islets. Islets were isolated (A) from 6 month-old male B6 WT and TG2^−/−^ (i) or 129 WT and TG2^−/−^ (ii) mice fed a normal chow diet (chow) or fed a normal chow diet for 3 months followed by a high-fat diet for 3 months (fat) or (B) from 3 month old male 129 WT and TG2^−/−^ mice fed a normal chow diet. (A) Isolated islets, 5 per group (n = 7), were incubated in Krebs-Ringer bicarbonate (KRB) buffer containing 2.8 mM glucose or 16.8 mM glucose or 16.8 mM glucose plus 0.1 mM carbachol, 37°C, 1 h. (B) Groups of 70 islets (n = 3 for WT and n = 2 for TG2^−/−^) were perifused in KRB containing 2.8 mM glucose for 10 min before a stimulatory period of 60 min with KRB containing 16.8 mM glucose. The perifusate was switched back to 2.8 mM glucose for 30 min to allow insulin secretion to return to basal levels before exposure to 16.8 mM glucose and 100 µM carbachol for 30 min. The perifusate was switched back to 2.8 mM glucose for 10 min before non-metabolic depolarisation with 24 mM KCl to activate voltage-gated Ca^2+^ channel-triggered insulin secretion. One min fractions of the perifusate were collected for the first 10 min, then 2 min fractions were collected at a flow rate of 0.5 ml/min. Insulin content of supernatant and of islets was determined by radioimmunoassay. Data are presented as means ± SEM. Overall *P* values calculated using 2-way ANOVA are shown at the top; individual Bonferroni *post hoc* test results are shown above the columns.**P<0.01; ***P<0.001.

To further evaluate a role for TG2 in GSIS or in muscarinic potentiation of GSIS, we isolated islets from 6 month-old congenic male B6 or 129 WT and TG2^−/−^ littermates fed normal chow for 6 months or fed normal chow for 3 months followed by a high-fat diet for 3 months. Islets were analysed for GSIS and cholinergic potentiation of insulin secretion. We found no difference in total insulin content per islet between B6 WT and TG2^−/−^ or 129 WT and TG2^−/−^ mice (Table 1). The fraction of islet insulin content secreted after 1 h incubation with 2.8 mM glucose, 16.8 mM glucose or 16.8 mM glucose plus 0.1 mM carbachol was no different between islets from B6 WT and TG2^−/−^ mice or between 129 WT and TG2^−/−^ mice fed the standard chow diet (Fig. 3A). Consistent with the weight gain (Table 1) and elevated serum insulin levels (Fig. 2) observed in fat-fed B6 mice, but not in 129 mice, relative to their chow-fed controls, B6 WT and TG2^−/−^, but not 129 WT and TG2^−/−^ islets isolated from fat-fed mice released a higher fraction of their insulin content than those isolated from chow-fed mice when incubated with 16.8 mM glucose or 16.8 mM glucose plus 0.1 mM carbachol (Fig. 3A). As expected from WT islets isolated from mice in either dietary group, the fraction of islet insulin content secreted in response to incubation with 16.8 mM glucose was increased significantly relative to incubation with 2.8 mM glucose, and GSIS was potentiated in the presence of carbachol (Fig. 3A). Surprisingly, there was no significant difference between WT and TG2^−/−^ islets isolated from mice of the same congenic background and in the same dietary group with respect to the fraction of insulin content secreted in response to 2.8 mM glucose or 16.8 mM glucose or 16.8 mM glucose plus 0.1 mM carbachol (Fig. 3A). These data thus demonstrate that 6 month-old male B6 mice are significantly more responsive than similarly-aged 129 mice in glucose homeostasis and dietary effects on this homeostasis, but reveals no significant difference in GSIS or muscarinic potentiation of GSIS between congenic WT and TG2^−/−^ mice.

Lack of TG2 involvement in GSIS was further confirmed by more sensitive perifusion analysis, which showed that GSIS and carbachol potentiation of GSIS were completely unaltered in TG2^−/−^ relative to WT mice (Fig. 3B).

### Intraperitoneal Glucose Tolerance Tests in WT and TG2^−/−^ Mice

**Figure 4:**
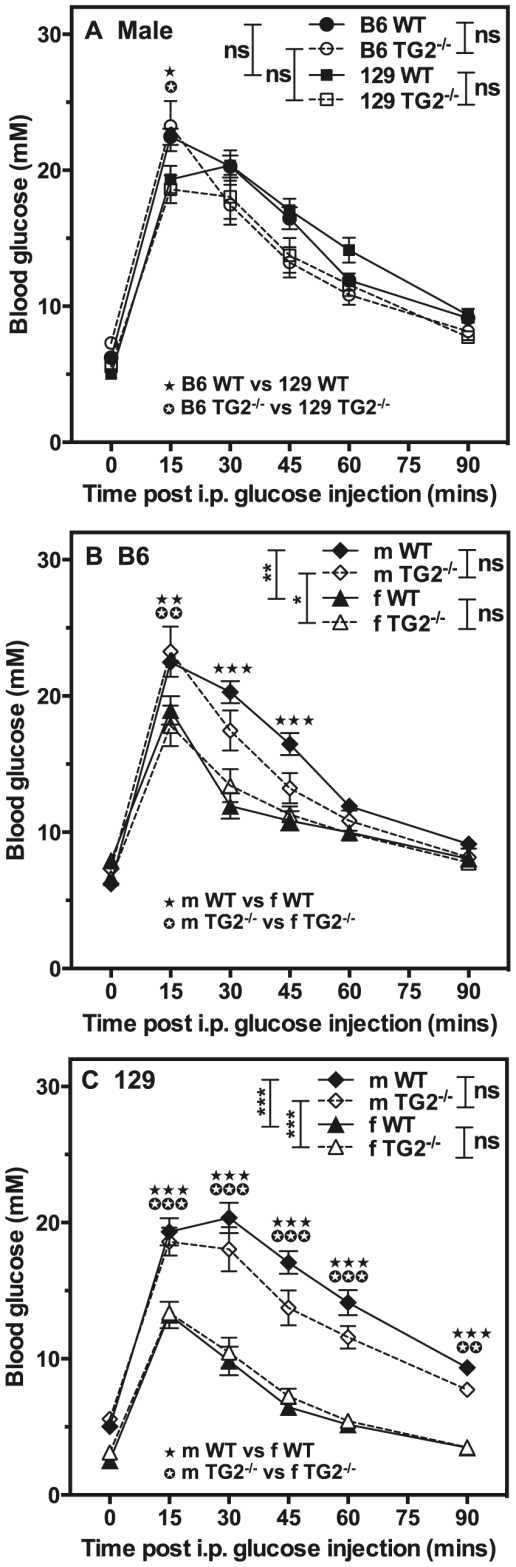
*Tgm2* deletion has no effect on intraperitoneal glucose tolerance tests in 3 month-old male or female mice. Fasted male or female 129 WT and TG2^−/−^ or B6 WT and TG2^−/−^ mice were subjected to intraperitoneal (i.p.) glucose tolerance tests and blood glucose levels were determined. Data are presented as means ± SEM (n = 6–10). Overall *P* values calculated using 2-way ANOVA are shown at the top; individual Bonferroni *post hoc* test results are shown above the line profiles.*P<0.05; **P<0.01; ***P<0.001.

Since TG2 had originally been implicated in glucose tolerance [3] as a result of intraperitoneal glucose tolerance testing, which does not involve vagal amplification of insulin secretion, we also attempted to confirm this phenotype in our congenic lines by investigating the responses of 3 month-old B6 or 129 WT and TG2^−/−^ littermates to intraperitoneal delivery of a glucose bolus. At this age, unlike at 6 months of age, average fasting body weights were not markedly different between male B6 and 129 mice, whereas female 129 mice had significantly lower average fasting body weights (p<0.001) than their B6 counterparts (Table 2). Fasting blood glucose levels were not markedly different between B6 WT and TG2^−/−^ and 129 WT and TG2^−/−^ mice, except for those of female 129 WT mice, which were significantly lower (p<0.001) than those of female B6 WT mice (Table 2). In response to intraperitoneal glucose delivery, there was no difference in blood glucose levels between male congenic B6 and 129 WT and TG2^−/−^ mice (Fig. 4A), or between female congenic B6 (Fig. 4B) or 129 (Fig. 4C) WT and TG2^−/−^ mice. There was, however, a striking difference in intraperitoneal glucose tolerance between congenic B6 (Fig. 4B) or 129 (Fig. 4C) males and females, with males being less glucose tolerant than females, as indicated by their higher and more prolonged increases in blood glucose (Fig. 4B, C). Thus, our results, using two different congenic TG2 knock-out lines, indicate that glucose homeostasis is unaltered by TG2 deletion.

**Table 2:** Fasting body weights (g), blood glucose (mM) and serum insulin (ng/ml) concentrations in 3–4 month-old mice.

		Male			Female		
Genetic background	Genotype	Body weight	Blood glucose	Serum insulin	Body weight	Blood glucose	Serum insulin
B6	WT	22.0±0.4	6.2±0.3	0.29±0.05	24.1±0.4	7.9±0.5	0.21±0.01
	TG2^−/−^	24.7±0.3	7.3±0.3	0.28±0.03	23.9±0.8	6.6±0.3	0.21±0.04
129	WT	21.1±0.4	5.0±0.3	ND	16.7±0.7‡	2.5±0.2‡	ND
	TG2^−/−^	20.9±1.1	5.6±0.2	ND	15.6±0.3‡	3.1±0.1	ND
B6	WT	26.0±1.0	8.2±0.5	0.30±0.04	24.1±0.3	7.9±0.5	0.17±0.01
	TG2^R579A/R579A^	28.3±1.2	9.8±0.5	0.27±0.03	20.8±0.2*	7.3±0.4	0.20±0.02

Values are presented as means ± SEM (n = 6–11).

* *P*<0.001 vs male;

‡ *P*<0.001 vs corresponding B6 genotype.

### Generation and Characterisation of TG2^R579A/R579A^ Mice with Constitutively Active Transamidase Activity

**Figure 5:**
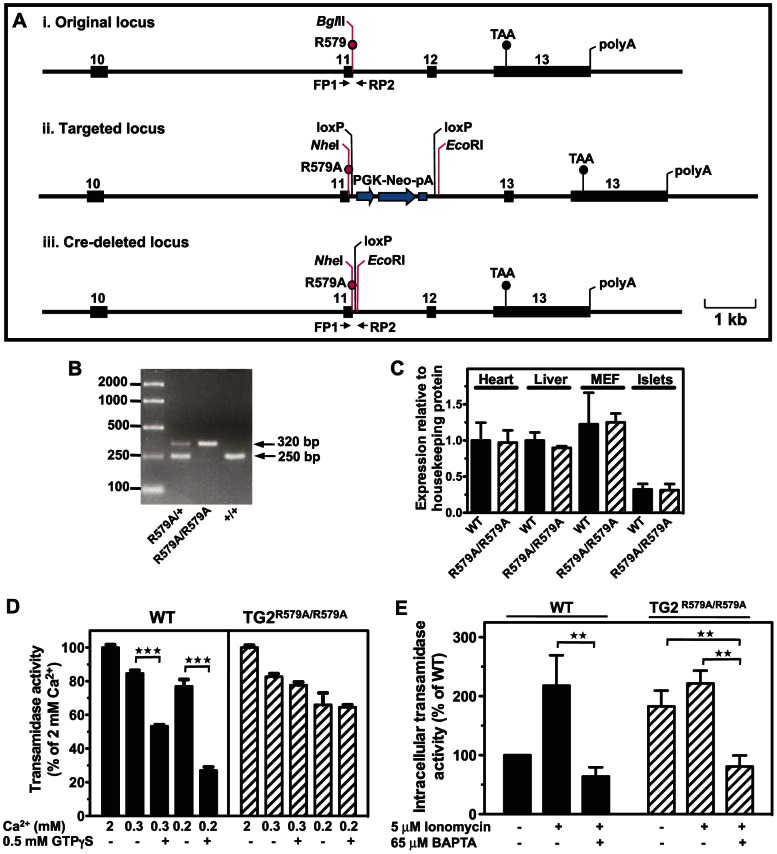
Generation of coisogenic B6 *Tgm2^R579A^* knock-in mice constitutively active for transamidating activity. A. Gene targeting strategy. i. Original locus showing location of Arg579 and *Bgl*II site (red line) in exon 11 (black box) of *Tgm2*. PCR primers, FP1 & RP2, yield a 250 bp *Bgl*II-cleavable fragment. ii. Targetted locus showing *Tgm2^R579A^* mutation, introduced *Nhe*I site, and insertion of a LoxP-flanked PGK–neomycin resistance selection cassette between exons 11 & 12. iii. Cre-deleted locus showing *Tgm2^R579A^* mutation, introduced *Nhe*I site, and remnant loxP site. PCR primers, FP1 & RP2, yield a 320 bp *Nhe*I-cleavable fragment. B. PCR primers, FP1 and RP2, distinguish the 250 bp wild-type *Tgm2* allele from the 320 bp *Tgm2^R579A^* knock-in allele. C. Homogenized tissues from WT or TG2^R579A/R579A^ mice (n = 3) were size-fractionated and TG2 levels detected by Western blot were normalized for loading using anti-GAPDH for heart, liver, MEF or tubulin for islets. D. GTPγS inhibition of *in vitro* calcium-activated transamidase activity was assayed in WT or TG2^R579A/R579A^ liver lysates (n = 3) as described in “Materials and Methods”. Transamidase activity was maximal (100%) with addition of 2mM CaCl_2_. *P* values were calculated using 1-way ANOVA. E. Intracellular transamidase activity was assayed in intact WT or TG2^R579A/R579A^ murine embryonic fibroblasts (n = 6) as described in “Materials and Methods”. *P* values were calculated using 2-way ANOVA with the Bonferroni *post hoc* test. Data are presented as means ± SEM. **P<0.01; ***P<0.001.

Gene knock-out experiments analyse the effect of the missing gene product rather than the effect of the gene product directly. To directly study the role of the transamidase activity of TG2 in insulin secretion, we generated a mouse (Fig. 5) that expresses a mutant form of TG2 (TG2^R579A^), which displays constitutively active transamidase activity [29]. Given the concurrence of our results with the established literature that B6 is a more suitable genetic background than 129 for phenotypic analyses of diabetogenic diseases, the TG2^R579A/R579A^ line was established on the B6 background (Fig. 5A, B). qRT-PCR (Fig. 1) and Western blot (Fig. 5C) analyses indicated that TG2^R579A^ mRNA and protein levels in a range of TG2^R579A/R579A^ tissues and primary cells were equivalent to those of their WT littermates, with no change in expression of other TGs. We have shown previously that calcium activates TG2 transamidase activity and that GTP binding inhibits transamidase activity at calcium concentrations submaximal for transamidase activation [28]. We have also shown that Arg^579^ is important for GTP binding by TG2 and that loss of GTP binding by TG2^R579A^ abolishes GTP regulation of transamidase activity in intact cells, resulting in disinhibition, or constitutive activation, of intracellular transamidase activity [29]. Consistent with our previous work, *in vitro* calcium-activated transamidase activity of TG2^R579A^, but not WT TG2, was insensitive to GTPγS inhibition (Fig. 5D), and basal intracellular transamidase activity of TG2^R579A^ in intact cells was elevated relative to WT TG2 (Fig. 5E). Stimulation with the calcium ionophore ionomycin increased WT TG2 transamidase activity to that of TG2^R579A^, but did not further stimulate TG2^R579A^ activity, indicating maximal and constitutive activation of TG2^R579A^ in the unstimulated state. Transamidase activity of both WT TG2 and TG2^R579A^ was inhibited by calcium chelation with BAPTA, confirming calcium dependence of the transamidase activity.

**Figure 6:**
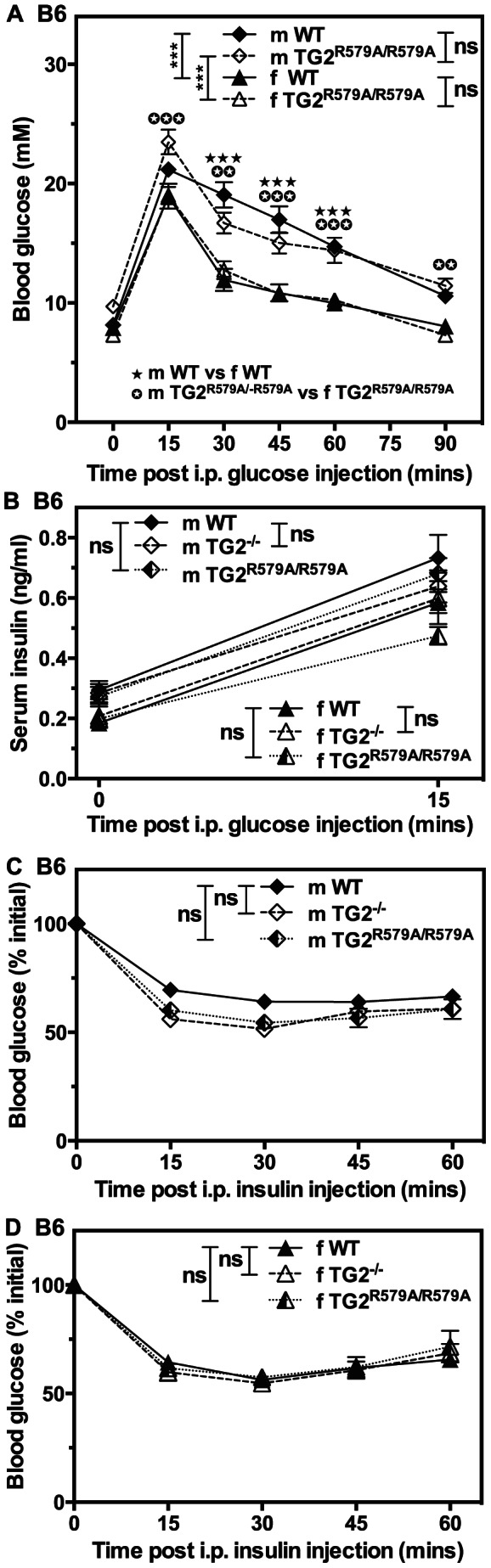
Neither *Tgm2* deletion nor substitution with *Tgm2^R579A^* affects glucose homeostasis in 3 month-old B6 mice. (A, B) Fasted B6 WT and TG2^R579A/R579A^ mice were subjected to intraperitoneal glucose tolerance tests or (C, D) fed B6 WT and TG2^−/−^ or B6 WT and TG2^R579A/R579A^ mice were subjected to intraperitoneal insulin tolerance tests, and blood glucose (A, C, D) or serum insulin levels (B) were determined. Data are presented as means ± SEM (n = 9–11). Overall *P* values calculated using 2-way ANOVA are shown at the top; individual Bonferroni *post hoc* test results are shown above the line profiles. **P<0.01; ***P<0.001.

Average body weights, fasting and non-fasting blood glucose levels, and fasting serum insulin levels of TG2^R579A/R579A^ mice were equivalent to those of WT littermates (Tables 2, 3). Intraperitoneal glucose tolerance tests revealed no difference in blood glucose (Fig. 6A) or serum insulin (Fig. 6B) levels between male or female WT and TG2^R579A/R579A^ mice. However, as observed previously, male mice were less glucose tolerant than female mice. Insulin tolerance tests (Fig. 6C, D) also revealed no significant differences between male or female WT, TG2^−/−^ and TG2^R579A/R579A^ mice.

**Table 3:** Randomly fed body weights (g) and blood glucose (mM) concentrations in 3–4 month-old mice.

		Male		Female	
Genetic background	Genotype	Body weight	Blood glucose	Body weight	Blood glucose
B6	WT	29.1±0.8	9.3±0.3	20.7±0.4*	8.1±0.3
	TG2^−/−^	27.8±0.5	10.4±0.2	20.2±0.4*	8.5±0.3
B6	WT	27.9±0.5	10.7±0.5	21.0±0.3*	10.1±0.5
	TG2^R579A/R579A^	26.4±0.4	10.8±0.4	20.0±0.3*	9.1±0.2

Values are presented as means ± SEM (n = 9–11).

* *P*<0.001 vs male.

Taken together then, our data indicate that glucose homeostasis is not affected by either TG2 deletion or constitutive activation of TG2 transamidase activity.

## Discussion

The phenotype of glucose intolerance and impaired insulin secretion reported for the TG2^−/−^ mouse [3] was from a lineage derived from an undefined mix of both 129/SvJ and C57BL/6J (B6;129) parental genomes. It is well recognized by mouse geneticists that different inbred strains harbour different susceptibilities for Type 1 and Type 2 diabetes, and that components of parental backgrounds can contribute to metabolic dysfunction in undefined ways when a particular targeted gene mutation is on a segregating mixed background [33]. The 129 genome, for example, harbours latent, undefined diabetes susceptibility quantitative trait loci (QTL) capable of enhancing insulin resistance [33]. The influence of strain background on a phenotype is therefore of such potential significance that confirming a phenotype in two unrelated strains is highly desirable [34], [35]. Thus, to separate the specific metabolic contribution of TG2 deletion to diabetogenesis from undefined strain background contributions, our B6;129 TG2^+/−^ mice [6] were backcrossed to the B6 or 129 parental background to generate congenic (B6 or 129) heterozygous TG2^+/−^ mouse lines that are genetically identical to the parental strain background except for the genetically-modified allele and the chromosomal region immediately neighbouring it.

Using these two distinct congenic TG2 knock-out lines and a coisogenic knock-out/knock-in line, our results indicate that TG2 does not have a role in glucose homeostasis or in mediating muscarinic modulation of glucose homeostasis. The lack of a significant difference in glucose homeostasis between the 129 or B6 WT and TG2^−/−^ mice and the B6 WT and TG2^R579A/R579A^ mice described here is in itself reassuring because it indicates that the targeted embryonic stem cells (which were injected into blastocysts to generate the original TG2 knock-out or TG2 knock-out/knock-in founder mice) did not have an accumulation of culture-induced chromosomal aberrations upstream or downstream of the disrupted allele that might generate phenodeviants independent of the targeted mutation. Moreover, RT-qPCR analyses indicated a lack of compensatory enhanced expression of other TGs in these mice.

Our data, however, are contrary to prior reports implicating a role for TG2 in insulin secretion [3], [18]–[21]. One of these studies [3] used mixed-strain TG2^−/−^ mice generated independently by another group [5]. As shown by us here and reported elsewhere, both the B6 and 129 parental strains show normal insulin sensitivity. However, the phenomenon of unanticipated insulin resistance being generated when two unrelated genomes are combined, is well known [33]. Thus, it is possible that unexpected contributions from the 129 genome that interact deleteriously with the B6 genome might explain the glucose tolerance and impaired insulin secretion reported by Bernassola *et al*., (2002) using their mixed-strain TG2^−/−^ mice. Another study [21] recently reported impaired insulin secretion by islets isolated from that same TG2^−/−^ mouse line, now reported to be on a B6 background, although the number of generations of backcrossing and, thus, the genomic homogeneity of the line was not stated. Other studies implicating transamidase activity in insulin secretion [18]–[20] have used small molecule inhibitors such as methylamine, cystamine and dansylcadaverine, which, in addition to being alternate substrates that competitively inhibit transglutaminases [36], are well-known to have many biological effects, including antioxidant activity, inhibition of a range of cysteine proteases, as well as inhibition of endocytosis [37]–[39]. Their mode of action, therefore, cannot be considered to be so restricted as to interfere with transglutaminases exclusively [36].

Three different missense mutations (M330R, I331N, N333S) that impair transamidase activity have been found in TGM2 in 3 families with early-onset Type 2 diabetes [3], [4]. A single heterozygous missense mutation (c.998A>G, p.N333S) was identified in an Italian individual with MODY and in his diabetic father [3]; a second heterozygous missense mutation (c.989T>G, p.M330R) was identified in a Danish patient but not in his dizygotic twin or mother, and a third heterozygous missense mutation (c.992T>A, p.I331N) was identified in two diabetic siblings and two siblings with intermittent impaired fasting glucose, but not in another sibling with diabetic neuropathy [4]. Thus, although these mutations have not been found in 600 normoglycaemic controls, the heterozygous TGM2 mutations are not fully penetrant and do not appear to be the only cause of diabetes in these families [4]. Given that, in the mice generated in this study, neither deletion nor constitutive activation of TG2 transamidase activity altered basal responses or responses upon glucose or insulin challenge, our results call into question the link between TG2 and diabetes reported in some human studies, although we cannot exclude a possible species difference.
